# Open-wedge high tibial osteotomy with a slight valgus correction from neutral limb alignment achieves clinical improvements comparable with those for knees with varus deformity

**DOI:** 10.1186/s40634-023-00640-w

**Published:** 2023-07-29

**Authors:** Junya Itou, Umito Kuwashima, Masafumi Itoh, Ken Okazaki

**Affiliations:** Department of Orthopaedic Surgery, Tokyo Women’s Medical University, 8-1 Kawada-Cho, Shinjuku-Ku, Tokyo, 162-8666 Japan

**Keywords:** High tibial osteotomy, Alignment, Patient-reported outcome measures, Neutral alignment, Minimum clinically important difference

## Abstract

**Purpose:**

The effect of open-wedge high tibial osteotomy (OWHTO) on the preoperative neutral alignment of the knee is unknown. The purpose of this study was to clarify the clinical outcome of OWHTO with neutral alignment, defined as within 4 degrees of varus.

**Methods:**

This retrospective study included 72 knees with varus that underwent medial OWHTO. The knees were divided according to the preoperative hip-knee-ankle angle into a neutral alignment group (≤ 4° of varus alignment) and a varus alignment group (> 4° of varus alignment). The Knee Injury and Osteoarthritis Outcome Score (KOOS) and the Forgotten Joint Score-12 (FJS-12) were evaluated preoperatively and during at least 2 years of follow-up postoperatively.

**Results:**

There were no significant differences between the preoperative FJS-12 (17.9 versus 23.7; *p* = 0.16) and postoperative FJS-12 (57.3 versus 60.6; *p* = 0.52) or KOOS subscale scores (*p* > 0.05) in the neutral alignment group or the varus alignment group. Each group had a mean change in the KOOS subscale scores that exceeded the minimum clinically important difference.

**Conclusion:**

The short-term clinical results of OWHTO for neutral alignment were as favourable as those for varus malalignment.

**Level of evidence:**

IV.

## Introduction

Medial open-wedge high tibial osteotomy (OWHTO) is widely known for its favourable outcomes when used to treat varus knees with osteoarthritis (OA) or osteonecrosis [3, 7, 16]. It is also widely known that lateral closed-wedge high tibial osteotomy or double-level osteotomy may be indicated when the degree of varus is severe [16, 23–26]. However, when the degree of varus is slight, the surgical indications may be an issue because of overlap between the indications for OWHTO and those for unicompartmental knee arthroplasty [4]. Moreover, OWHTO may be a better option for those who are younger and more active physically [4]. Although OWHTO is a satisfactory surgical treatment for symptomatic medial knee OA, especially in its relatively early stages with varus knee alignment, a number of surgeons have been concerned about how to treat patients with medial degenerative disease and neutral or slightly varus knee alignment [13]. OWHTO for neutral or slightly varus alignment requires only a few degrees of valgus correction, and it is not clear whether such a slight change in limb alignment would achieve clinically significant improvement.

Various definitions of slight varus of the knee have been reported, but no clear consensus has been reached. According to Sterett and Steadman [27], less than 5 degrees in the weight-bearing axis could be considered well aligned, while Sauerschnig et al. defined varus of less than 1 degree as normal [25]. Several comprehensive classifications of lower limb alignment have recently been proposed [9, 18, 19], although no consensus has yet been reached on a definition of neutral alignment. Therefore, the clinical outcome of OWHTO for neutral alignment is not known.

The purpose of this study was to clarify the clinical outcome of OWHTO with neutral alignment when defined as within 4 degrees of varus. It was hypothesised that OWHTO for neutral alignment would have an outcome similar to that of OWHTO for varus alignment.

## Materials and methods

This retrospective study included 75 varus knees that underwent primary OWHTO between April 2017 and April 2021 at Tokyo Women’s Medical University. OWHTO was performed in patients who complained of anteromedial knee pain after at least 3 months of conservative treatment and had accompanying medial degenerative disease such as radiographic medial OA, medial osteonecrosis, degenerative medial meniscus tear with bone marrow lesions in the medial compartment or posterior root tear of the medial meniscus. At our institution, closed-wedge high tibial osteotomy was indicated for knees requiring more than 12° valgus correction.

**Fig. 1:**
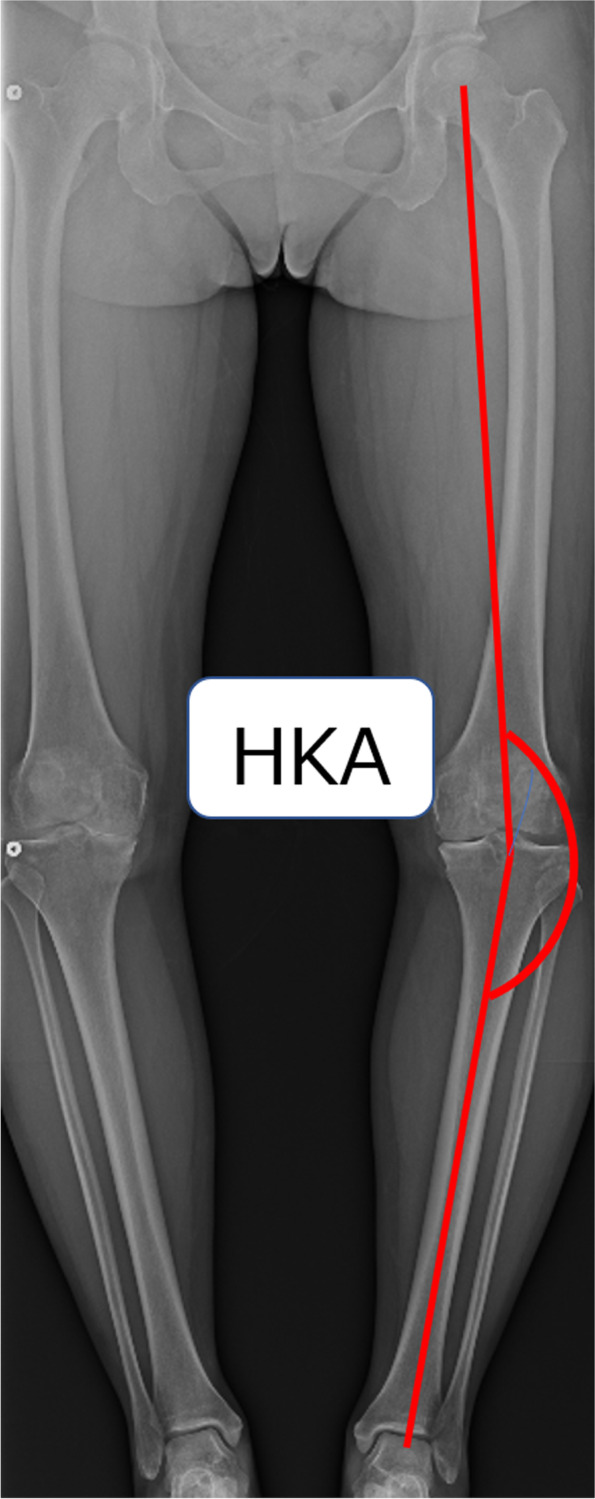
Measurement of the HKA on whole-leg standing radiographs. HKA, hip-knee-ankle angle

The inclusion criteria were patient-reported outcome measures (PROMs) evaluated preoperatively and postoperatively during at least 2 years of follow-up and preoperative and postoperative full-length weight-bearing radiographs available. The exclusion criteria were moderate to severe degenerative changes in the lateral or patellofemoral compartment that the attending surgeon considered unacceptable, incomplete PROMs data and inadequate radiological data. After exclusions, this left 72 knees for analysis. The knees were divided according to the preoperative hip-knee-ankle (HKA) angle measured on digital long-leg standing radiographs into a neutral alignment group (≤ 184° of varus alignment) and a varus alignment group (> 184° of varus alignment). The HKA angle was defined as the angle formed by a line drawn from the centre of the femoral head to the centre of the knee and a line drawn from the centre of the knee to centre of the ankle, as shown in Fig. 1.

The study protocol was approved by our institutional ethics committee of Tokyo Women’s Medical University (approval number: 4578). Informed consent was obtained via the opt-out method.

### Surgical technique and rehabilitation

**Fig. 2:**
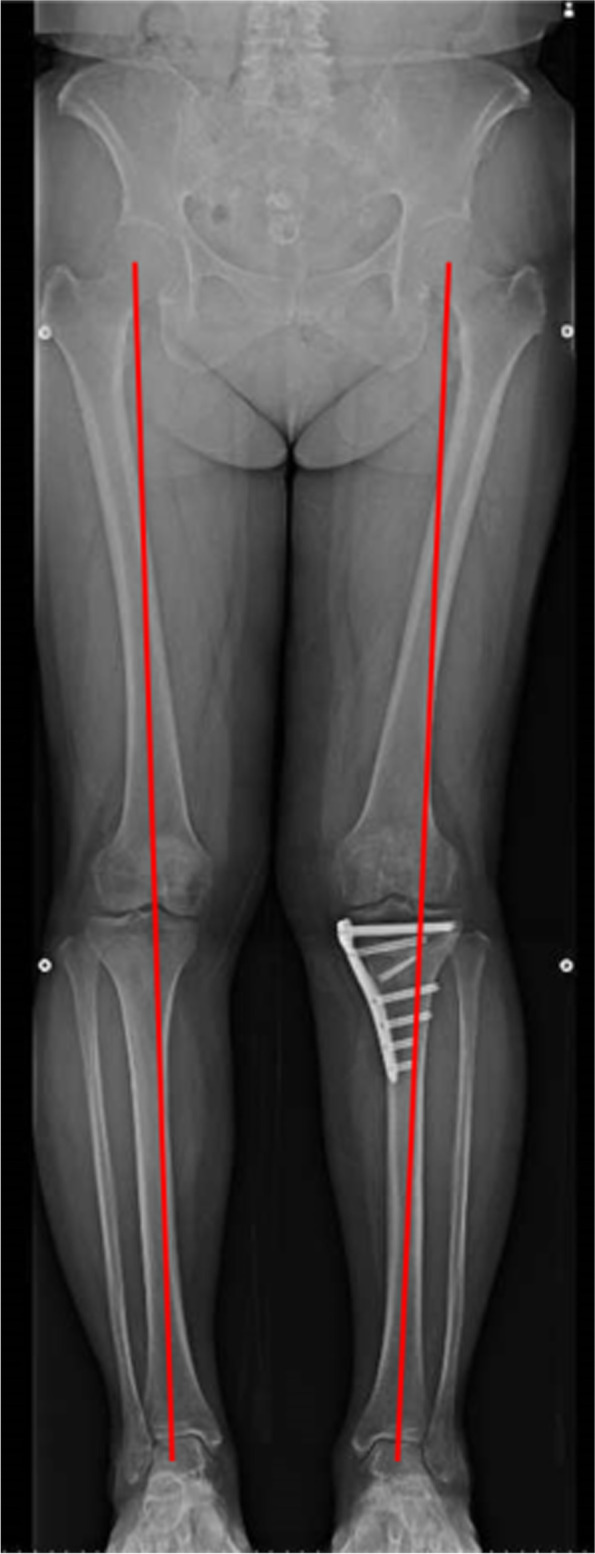
Radiographs after open-wedge high tibial osteotomy for neutral alignment. The red lines represent the mechanical axis of the lower limb on standing whole-leg radiograph. The correction angle of the left knee was 6 degrees

All surgical procedures were performed by one of four experienced knee surgeons. HTO was performed by the medial OWHTO method [10–13] using a long locking plate (TriS, Olympus Terumo Biomaterials, Tokyo, Japan). The correction angle was calculated using the method described by Miniaci et al. [21] The target point, known as the Fujisawa point [5], which is 62.5% of the overall width of the tibial plateau measured from the medial side, was set (Fig. 2). Artificial bone (OSferion 60, Olympus Terumo Biomaterials) was inserted into the osteotomy gap in all cases.

All patients were allowed to start partial weight-bearing using double crutches 1 week after surgery. Full weight-bearing was allowed after 3 weeks.

### Arthroscopic findings and concurrent procedures

A standard arthroscopic evaluation was carried out through standard anteromedial and anterolateral portals. Concurrent arthroscopic resection or repair of a torn medial meniscus was performed using an all-inside meniscus suture device at the discretion of the surgeon. Cartilage repair or a pull-out repair for a torn posterior root of the medial meniscus was not performed in this series. In addition, the status of the anterior cruciate ligament was normal or frayed in all cases, with no cases classified as disrupted or absent.

### Radiological parameters and PROMs

The HKA was measured preoperatively and postoperatively on whole-leg standing radiographs (Fig. 1). The Kellgren-Lawrence OA grade was evaluated on preoperative plain radiographs. Intraobserver reliability of measurements was assessed using the intraclass correlation coefficient (ICC). Measurements were repeated after a 2-week interval to evaluate the ICCs for the radiological parameter (HKA) in 20 knees. The ICC for intraobserver agreement regarding radiological HKA was 0.90.

Patient data, including age, sex and preoperative body mass index, were collected from the medical records. To evaluate PROMs, patients were asked by the attending surgeon to complete the Knee Injury and Osteoarthritis Outcome Score (KOOS), University of California, Los Angeles activity score questionnaire and Forgotten Joint Score-12 (FJS-12) preoperatively and postoperatively. The minimum clinically important difference (MCID) for preoperative and postoperative changes in the KOOS subscale scores following OWHTO were as follows: pain, 15.4; symptoms, 15.1; activities of daily living, 17.0; sport, 11.2; and quality of life 16.5 [14].

### Statistical analyses

Descriptive statistics are reported as median (range), number (percentage) or mean and standard deviation. The distribution of continuous variables was assessed for normality by visual inspection of histograms and using the Shapiro–Wilk test. Differences between the two alignment groups were examined using the chi-square test for categorical variables and the Wilcoxon signed-rank test for continuous variables. A post hoc power analysis was conducted using G*Power (version 3.1.9.7). Based on an effect size of 0.5, a total sample size of 72 and an alpha error probability of 0.05 for two groups, it was calculated that a power of 0.88 would be required. All statistical analyses were performed using JMP software version 16 (SAS Institute Inc., Cary, NC, USA). A *p*-value < 0.05 was considered statistically significant.

## Results

**Fig. 3:**
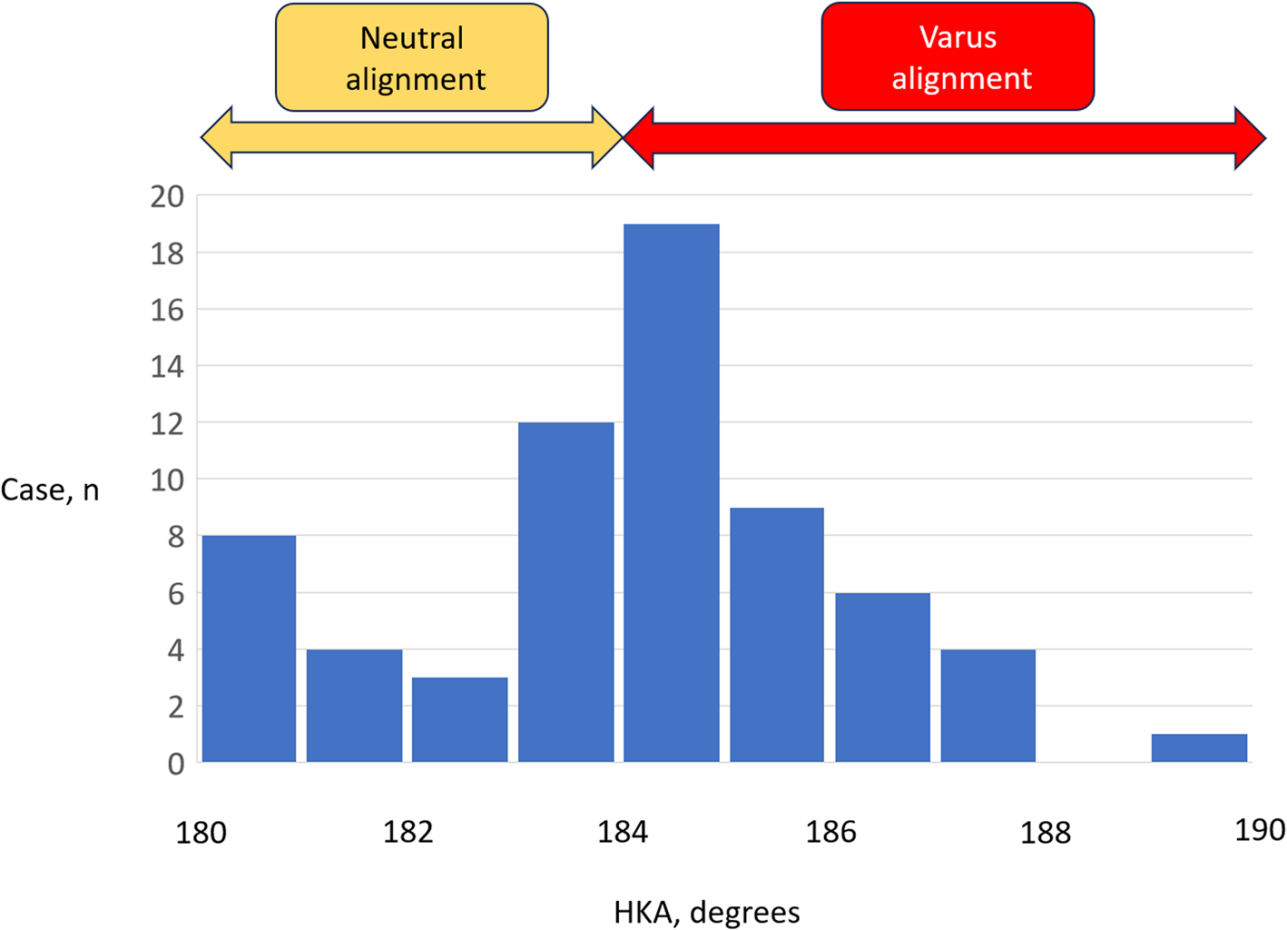
Histogram showing preoperative degree of varus alignment. The knees were divided according to the preoperative HKA angle measured on digital long-leg standing radiographs into a neutral alignment group (≤ 184° of varus alignment) and a varus alignment group (> 184° of varus alignment). HKA, hip-knee-ankle angle

Patient demographic and radiological data according to alignment group are shown in Table 1. Twenty-seven of the 72 knees showed neutral alignment (Fig. 3). Mean age was significantly younger in the neutral alignment group. There was a significant difference in the correction angle because the target alignment of the valgus osteotomy and the postoperative alignment was the same between the groups (Table 1). In addition, there was no significant difference in concurrent arthroscopic procedures between the two groups.

**Table 1:** Demographic and radiological data

	Neutral alignment (*n* = 30)	Varus alignment (*n* = 42)	*p*-value
Age, years, mean ± SD	54.8 ± 5.7	59.6 ± 8.6	**0.01**
Male sex, n (%)	12 (40.0)	18 (42.8)	1.00
K/L grade, 1, 2, 3, 4	16, 8, 6, 0	18, 12, 10, 2	0.68
Body mass index, mean ± SD	25.4 ± 4.2	25.5 ± 4.0	0.78
Right side affected, n (%)	15 (50.0)	18 (42.8)	0.63
Preoperative ROM, (°), mean ± SD	132.6 ± 15.4	134.3 ± 10.9	0.95
Postoperative ROM, (°), mean ± SD	142.2 ± 7.0	142.4 ± 5.4	0.83
Follow-up, months, median [range]	24.0 [24.0–41.3]	26.0 [24.0–36.0]	0.66
Preoperative UCLA, median [range]	6 [4-9]	6 [4.75–8]	0.95
Preoperative HKA, mean ± SD	182.3 ± 1.2	185.5 ± 1.3	**< 0.0001**
Postoperative HKA, mean ± SD	176.5 ± 1.2	177.0 ± 1.5	0.08
Correction angle, mean ± SD	5.7 ± 1.8	8.5 ± 1.8	**< 0.0001**
Concurrent arthroscopic procedures, n (%)	Meniscus repair: 9 (30.0) Meniscus resection: 21 (70.0)	Meniscus repair: 10 (23.8) Meniscus resection: 32 (76.2)	0.59

*HKA* Hip-knee-ankle angle, *K/L* Kellgren-Lawrence, *ROM* Range of motion, *SD* Standard deviation, *UCLA* University of California, Los Angeles

Bold values denote statistically significant differences between the alignment groups

For PROMs, there was no significant difference in the preoperative or postoperative FJS-12 or KOOS subscale scores between the two groups (Table 2). Each group had a mean change in the KOOS subscale score that exceeded the MCID (Table 3).

**Table 2:** Comparison of patient-reported outcome measures

	Preoperative			Postoperative		
	Neutral alignment (*n* = 30)	Varus alignment (*n* = 42)	*p*-value	Neutral alignment (*n* = 30)	Varus alignment (*n* = 42)	*p*-value
FJS-12	17.9 ± 13.8	23.7 ± 17.6	0.16	57.3 ± 28.1	60.6 ± 28.4	0.52
KOOS						
Pain	55.0 ± 16.4	53.9 ± 17.8	0.89	83.8 ± 15.3	87.5 ± 12.7	0.35
Symptoms	60.0 ± 19.9	58.1 ± 17.7	0.51	85.2 ± 12.8	86.6 ± 14.5	0.45
ADL	68.1 ± 17.2	66.5 ± 17.2	0.56	87.3 ± 13.8	90.7 ± 11.6	0.40
Sports	32.1 ± 22.5	32.7 ± 24.3	0.97	66.7 ± 26.8	70.5 ± 24.2	0.65
QOL	28.5 ± 19.5	31.7 ± 18.5	0.40	70.2 ± 23.0	70.8 ± 22.4	0.97

Data are shown as the mean ± standard deviation

*ADL* Activities of daily living, *FJS-12* Forgotten Joint Score, *KOOS* Knee Injury and Osteoarthritis Outcome Score, *QOL* Quality of life

**Table 3:** Comparison of change in patient-reported outcome measures

	Neutral alignment (*n* = 30)	Varus alignment (*n* = 42)	*p*-value	MCID
ΔFJS-12	39.3 ± 29.9	36.9 ± 25.3	0.56	N/A
KOOS				
ΔPain	28.8 ± 18.4	33.2 ± 16.3	0.21	15.4
ΔSymptom	25.1 ± 22.7	28.0 ± 18.8	0.54	15.1
ΔADL	19.2 ± 15.5	23.8 ± 14.3	0.13	17.0
ΔSport	34.5 ± 27.8	36.6 ± 22.2	0.73	11.2
ΔQOL	41.6 ± 22.5	38.1 ± 22.8	0.47	16.5

Data are shown as the mean ± standard deviation

*ADL* Activities of daily living, *FJS-12* Forgotten Joint Score, *KOOS* Knee Injury and Osteoarthritis Outcome Score, *MCID* Minimum clinically important difference, *N/A* Not available, *QOL* Quality of life

## Discussion

The most important finding in this study was that the short-term clinical outcomes of OWHTO were favourable for both neutral alignment (≤ 184° of varus alignment) and varus malalignment (> 184° of varus alignment). This finding indicates that even slight change from neutral alignment of a limb achieves significant clinical improvement that exceeds the MCIDs for PROMs. Furthermore, the PROMs used in this study included the FJS-12, which has a low ceiling effect, as a comparator. However, a precise comparison was not intended because it is more critical for the treatment effect to be sufficient, even a slight change from neutral alignment of a limb, than for there to be no difference between the two groups.

Few studies have evaluated preoperative slight varus alignment, such as preoperative neutral alignment, following OWHTO. Furthermore, the definition of neutral alignment itself has not yet been standardised. Lin et al. [18] defined neutral alignment as HKA within 3 degrees, whereas Thienpont et al. [28] defined neutral mechanical alignment as HKA within 2 degrees. With varying definitions, preoperative neutral alignment has been reported in the range of 1–5 degrees to date [18, 19, 25, 27, 28]. Meanwhile, it has been suggested that the Fujisawa point is equal to about 3–5 degrees of mechanical valgus [6, 24, 29]. Hence, in the present study, if a valgus osteotomy was performed for small gaps of less than 9 degrees [2], less than 4 degrees of preoperative varus alignment was considered neutral alignment. Our study included 15 knees in which the HKA was within 3 degrees; these knees were defined as having neutral alignment. However, the results did not change when the neutral alignment group (≤ 183° of varus alignment) was compared with the varus alignment group (data not shown).

In this study, concurrent arthroscopic meniscus resection or repair was performed, and the effect of arthroscopic surgery cannot be excluded. However, there was no significant difference in concurrent arthroscopic procedures between the two groups. In addition, we did not perform pull-out repair for root tear of the medial meniscus in this series, and there are reports indicating that arthroscopic resection or repair with the all-inside devices does not have adjunctive clinical effects [8, 15, 17]. OWHTO combined with arthroscopic meniscal centralisation, not just simple suturing to the posterior root, has also been with reported [22]. Therefore, the relationship between more complex concurrent arthroscopic procedures and clinical outcomes requires further investigation.

Recently, MCID has been attracting attention as a method for evaluating PROMs. According to Jacquet et al. [14], the MCID value can indicate the effectiveness of a procedure. In our study, all KOOS subscale scores exceeded the MCID for neutral alignment. However, the ceiling effect of KOOS can be a concern in active patients, such as those who are candidates for OWHTO [10]. The FJS-12 was originally used to evaluate patients undergoing arthroplasty [1] and was recently validated for evaluation of OWHTO with a low ceiling effect [10]. The MCID for the FJS-12 value following OWHTO is still unknown and warrants investigation in future studies.

This study has several limitations. First, the sample size was small. Second, all patients were Asian, and the possibility of anatomical differences between ethnic groups was not evaluated. Third, the follow-up period was relatively short. Fourth, mean age was significantly different between the two alignment groups. This may have been influenced by surgical indications for the knee with neutral alignment, in that unicompartmental knee arthroplasty might have been used in elderly patients with advanced OA with neutral alignment. Moreover, it has been reported that knee surgeons frequently avoid joint arthroplasty in the young [20]. Finally, concurrent arthroscopic resection or repair of a torn medial meniscus was also a limitation. Although there were no differences in the meniscal procedures between the groups, the results of this study might be affected by these procedures.

Going forward, the present study and future work on the outcomes of OWHTO for neutral alignment will contribute to further treatment options for patients with medial degenerative disease and neutral or slightly varus knee alignment.

## Conclusion

The short-term clinical results of OWHTO were favourable for both neutral alignment and varus malalignment. This treatment method could be considered for slight varus malalignment of the knee.

## Abbreviations

FJS-12: Forgotten Joint Score-12
HKA: Hip-knee-ankle
ICC: Intraclass correlation coefficient
KOOS: Knee Injury and Osteoarthritis Outcome Score
MCID: Minimum clinically important difference
OA: Osteoarthritis
OWHTO: Open-wedge high tibial osteotomy
PROMs: Patient-reported outcome measures
